# Identification of Critical Biomarkers and Immune Infiltration in Rheumatoid Arthritis Based on WGCNA and LASSO Algorithm

**DOI:** 10.3389/fimmu.2022.925695

**Published:** 2022-06-29

**Authors:** Fan Jiang, Hongyi Zhou, Haili Shen

**Affiliations:** ^1^ Second Clinical Medical College, Lanzhou University, Lanzhou, China; ^2^ Department of General Medicine, Beijing Luhe Hospital, Capital Medical University, Beijing, China; ^3^ Department of Anesthesiology, Tongzhou Maternal and Child Health Hospital of Beijing, Beijing, China; ^4^ Department of Rheumatology, Lanzhou University Second Hospital, Lanzhou, China

**Keywords:** rheumatoid arthritis, diagnostic marker, machinelearning, GEO datasets, immune cells infiltration

## Abstract

Rheumatoid arthritis(RA) is the most common inflammatory arthritis, and a significant cause of morbidity and mortality. RA patients’ synovial inflammation contains a variety of genes and signalling pathways that are poorly understood. It was the goal of this research to discover the major biomarkers related to the course of RA and how they connect to immune cell infiltration. The Gene Expression Omnibus was used to download gene microarray data. Differential expression analysis, weighted gene co-expression network analysis (WGCNA), and least absolute shrinkage and selection operator (LASSO) regression were used to identify hub markers for RA. Single-sample GSEA was used to examine the infiltration levels of 28 immune cells and their connection to hub gene markers. The hub genes’ expression in RA-HFLS and HFLS cells was verified by RT-PCR. The CCK-8 assay was applied to determine the roles of hub genes in RA. In this study, we identified 21 differentially expressed genes (DEGs) in RA. WGCNA yielded two co-expression modules, one of which exhibited the strongest connection with RA. Using a combination of differential genes, a total of 6 intersecting genes was discovered. Six hub genes were identified as possible biomarkers for RA after a lasso analysis was performed on the data. Three hub genes, CKS2, CSTA, and LY96, were found to have high diagnostic value using ROC curve analysis. They were shown to be closely related to the concentrations of several immune cells. RT-PCR confirmed that the expressions of CKS2, CSTA and LY96 were distinctly upregulated in RA‐HFLS cells compared with HFLS cells. More importantly, knockdown of CKS2 suppressed the proliferation of RA‐HFLS cells. Overall, to help diagnose and treat RA, it’s expected that CKS2, CSTA, and LY96 will be available, and the aforementioned infiltration of immune cells may have a significant impact on the onset and progression of the disease.

## Introduction

Rheumatoid arthritis (RA) is a common chronic inflammatory joint disease characterized by persistent synovial hyperplasia and progressive destruction of joint cartilage and bone (1, 2). It is well recognized that RA can lead to decreased functional status, disability, and increased mortality (3). Around 1% of the population suffers from RA at any given time, and females are more likely than males to be affected (4, 5). The exact pathophysiology of RA is still not well understood. Studies have shown that it may be linked to immune system variables, environmental factors, genetics, and other factors (6, 7). Key aspects of RA’s pathogenesis, including lymphocyte infiltration and development of fibroblast-like synoviocytes (FLS) in the synovial fluid, have received major study attention (8, 9). Accordingly, it is imperative to investigate the molecular pathways that underlie the disease and find diagnostic biomarkers for RA in order to improve treatment outcomes for people with RA.

As more and more publicly available high-throughput data in worldwide were developed, an unanswered question has arisen: How can we leverage these large-scale data effectively to gain a full understanding of various diseases at the molecular levels (10, 11)? Human life is enriched by machine learning (ML), which is the scientific study of algorithms and statistical models (12). ML is particularly important in the identification of the potential biomarkers for the diagnosis and prognosis of human diseases, which is why it is being studied more and more in this sector (13, 14). A number of studies have used numerous markers to develop prediction models for early diagnosis in clinical patients, with mixed results (15, 16). However, prior researches have found that the accuracy of these models, which are comprised of predictive biomarkers, as well as their application scope, are significantly limited by the sample size (17, 18). The weighted gene co-expression network analysis (WGCNA) and the least absolute shrinkage and selection operator (LASSO) algorithms are widely used in bioinformatics analysis and exhibit an important in clinical application of various fields (19, 20). However, their application in screening potential biomarkers for RA was rarely reported.

In this investigation, we aimed to discover the major biomarkers related to the course of RA and how they connect to immune cell infiltration. We used two microarray datasets of RA that were retrieved from the GEO datasets. The study of differentially expressed genes (DEGs) was carried out between the RA and the controls. To filter and discover diagnostic biomarkers of RA, machine-learning techniques were applied. As a result of this study, for the first time, the fraction of immune cells in samples of RA and normal tissues was quantified using ssGSEA (single-sample gene set enrichment analysis). Moreover, we investigated the association between the biomarkers identified and the infiltrating immune cells in order to lay the groundwork for future studies.

## Materials and Methods

### Data Collection

The mRNA expression profile (Number: GSE17755 and GSE93272) was obtained from the GEO database (https://www.ncbi.nlm.nih.gov/geo/). GSE17755 contained blood samples of 99 RA patients and 45 healthy controls. GSE93272 contained blood samples of 232 RA patients and 43 healthy controls. The expression analysis of mRNA profile was detected by GPL1291 and GPL570.

### Cell Incubation and Transfection

HFLS and RA‐infected HFLS (RA‐HFLS) were obtained from Cell Applications, Inc. DMEM containing 10% fetal bovine serum, 1% penicillin/streptomycin, and 5% CO_2_ was used to keep the cells at 37°C in an incubator. Lipo 3000 transfection reagent(Thermo Fisher Scientific, MA, USA) was used to deliver the CKS2 siRNA (siCKS2) and its negative control into RAHFLS.

### Quantitative Real-Time PCR (qRT-PCR)

Based on manufacturer’s instructions, we extracted total RNA from cells using the TRIZOL reagent (Invitrogen, Carlsbad, CA, USA). The Reverse Transcription Kit was used to reverse-transcribe one microgram of total RNA into cDNA for use in the qRT-PCR assay (Takara, Dalian, China). With the use of the Fast Real-time PCR 7500 System(Applied Biosystems, Foster City, CA, USA), we were able to determine gene expression. After two minutes at 50°C, the PCR reaction was subjected to 40 cycles of 95°C for 15 seconds, followed by one minute at 60°C. The GAPDH gene was amplified to serve as an internal control. The relative quantification values for CKS2 were calculated by the 2^-ΔΔCt^ method. The primers were as follows: CKS2 sense: 5’-TTCGACGAACACTACGAGTACC-3’; CKS2 antisense: 5’- GGACACCAAGTCTCCTCCAC-3’; GAPDH sense: 5’-AGAAGGCT-GGGGCTCATTTG-3’; GAPDH antisense: 5’-AGGGGCCATCCACAGTCTTC-3’.

### Cell Proliferation Assay

Cells were harvested and detachable with 0.25 percent trypsin during the logarithmic growth phase. In 96-well plates, the cells were planted at a density of 2×10^3^ cells per well. Each well was incubated at 37°C for an additional 2 h after incubation for 0, 24, 48, 72 and 96 hours with sterile Cell Counting Kit-8 solution (15μL). Finally, an optical density (OD) value measurement at 450 nm was performed using a Thermo Multiskan MK3 reader (Thermo Fisher, Schwerte, Germany).

### Identification of Differentially Expressed Genes (DEGs)

It was normalised using RMA and the DEGs were evaluated using a limma R tool for GSE17755 dataset. Raw signals from the analysis were log2 transformed after quantile normalisation. [log2FC| > 1] and a false discovery rate of 0.05 were used to identify DEGs in this study.

### Construction of Gene Co-Expression Network

WGCNA is a bioinformatics analytical method that is used frequently to explore effectively the relationships between genes and phenotypes (21). The WGCNA tool in R was used to build a weighted co-expression network for the GSE17755 dataset’s expressing data before a subset of genes with absolute deviations greater than 25% from the median were selected for further investigation. The “goodSampleGenes” function was used to verify the data’s integrity. PickSoftThreshold was used to select and verify an optimum soft threshold (b). In order to find modules based on topological overlap, the matrix data were transformed into an adjacency matrix, and then clustered. Clustering dendrograms were generated after the computation of module eigengene (ME) and merging of related modules in the tree based on ME. Using phenotypic data and modules, the importance of genes and clinical data was assessed, and the relationship between models and modules was examined.

### Screening of the Critical Genes

Candidate hub genes were chosen from a pool of genes with the greatest degree of connection among modules. Absolute GS values tend to be greater in genes having biological importance. The criteria (absolute values of GS > 0.20 and MM > 0.80) were used to screen potential hub genes. LASSO is a regression-based methodology permitting for a large number of covariates in the model, and importantly has the unique feature penalizing the absolute value of a regression coefficient (22). In order to identify the final hub genes, we used the ‘glmnet’ package of R software to run LASSO analysis on the candidate hub genes and DEGs. Analysis of the levels of genes in RA samples and normal samples was carried out using box plots. The levels of hub genes that identify RA samples from healthy samples were assessed using ROC curves. In addition, a different dataset (GSE93272) was used to validate the levels of hub genes and diagnostic value.

### Immune Cells Infiltration Analysis

ssGSEA in the “GSVA” R package was used to analyse the immune infiltration of RA (23). Immune cells and hub gene expression were then correlated using Spearman’s correlation.

### Functional Enrichment Analysis

R packages “clusterProfiler” and “enrichplot” were used to perform GO assays, KEGG assays, and GSEA of DEGs with a statistically significant difference of at least P< 0.05 (24, 25). Gene sets with P 0.05 and a FDR q-value 0.05 were considered highly enriched in the MsigDB datasets for GSEA.

### Statistical Analysis

Statistical analyses and graphs were generated using GraphPad Prism version 5.0 (La Jolla, CA, USA) or R.4.1.1 (R Core Team, Massachusetts, USA). By using the Student t-test, we were able to determine the differences between groups. Hub genes’ diagnostic accuracy was tested using ROC curves. For all tests, p-values of < 0.05 were interpreted as statistically significant.

## Results

### Identification of DEGs in RA

**Figure 1:**
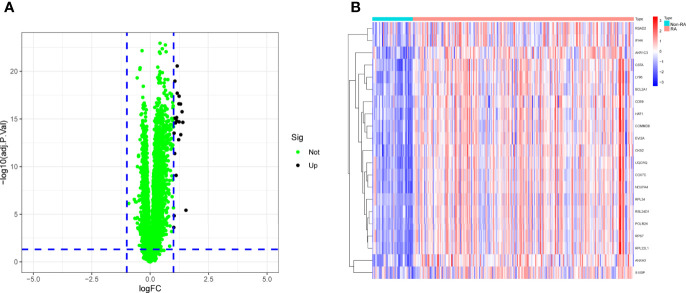
The dysregulated genes in RA from GSE17755 was shown in **(A) **Volcanic map and **(B) **Heat map.

To explore the possible biomarkers for RA, data from a total of 99 RA and 48 control samples from GSE17755 were retrospectively analyzed in this study. A total of 21 DEGs were discovered, and all of them showed significant increases in expressions (**Figures 1A, B**).

### Functional Enrichment Analysis of DEGs

**Figure 2:**
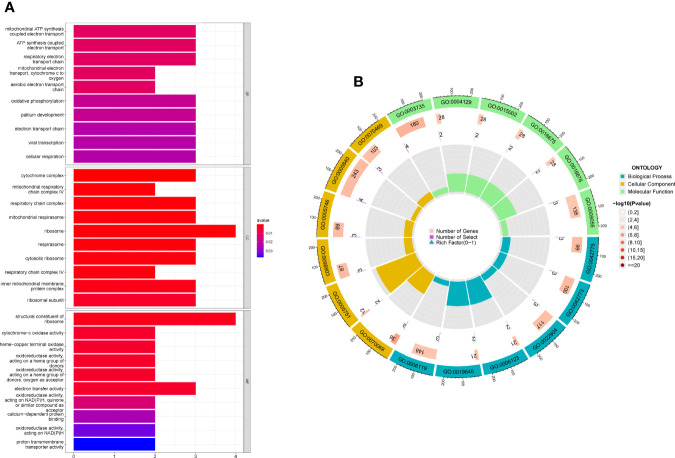
**(A, B) **GO term analysis of DEGs.

**Figure 3:**
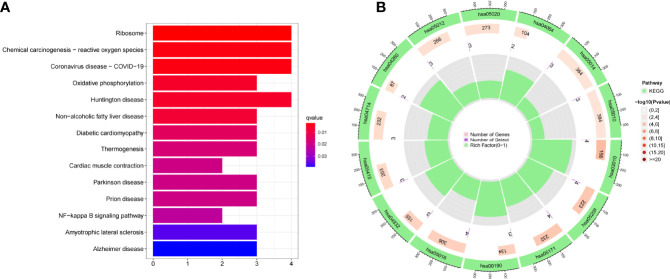
**(A, B) **KEGG term analysis of DEGs.

**Figure 4:**
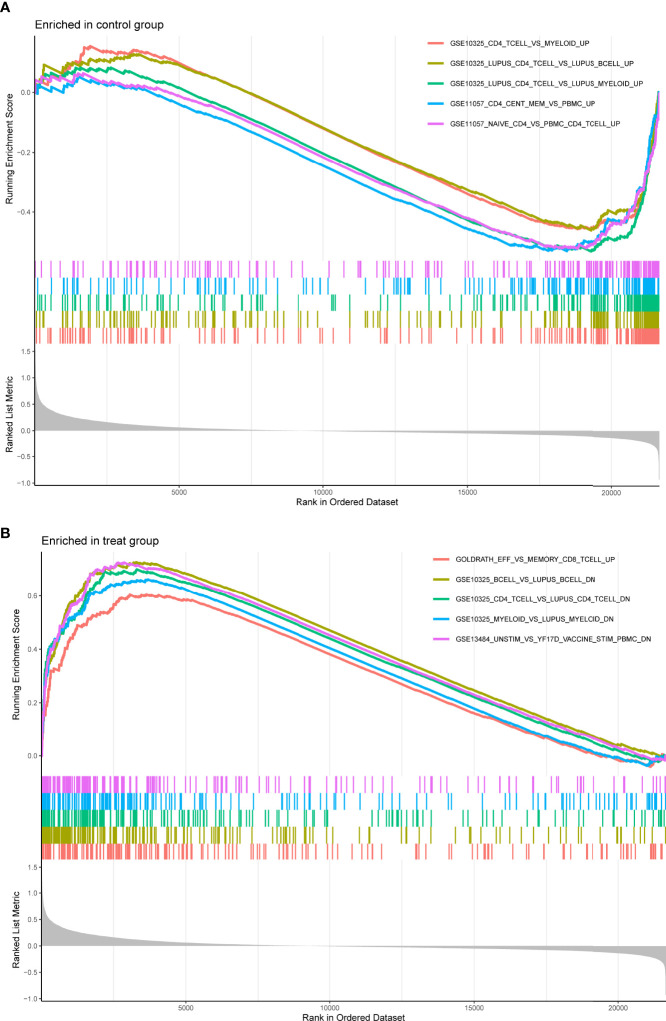
Enrichment analyses *via* gene set enrichment analysis. **(A)** Enriched in control group. **(B)** Enriched in treat group.

For a better understanding of the biological processes and signal pathways linked with RA DEGs, researchers used GO and KEGG analyses. The results of GO assays revealed that DEGs were mainly enriched in ATP synthesis coupled electron transport, mitochondrial ATP synthesis coupled electron transport, respiratory electron transport chain, cytochrome complex, mitochondrial respiratory chain complex IV, respiratory chain complex, structural constituent of ribosome, cytochrome-c oxidase activity and heme-copper terminal oxidase activity(**Figures 2A, B**). The outcomes of KEGG assays revealed that DEGs were mainly enriched in pathways involved in Ribosome, Chemical carcinogenesis-reactive oxygen species, Coronavirus disease- COVID-19, Oxidative phosphorylation and Huntington disease (**Figures 3A, B**). In addition, the results of GSEA assays were shown in **Figures 4A, B**.

### Screening and Verification of Diagnostic Markers

**Figure 5:**
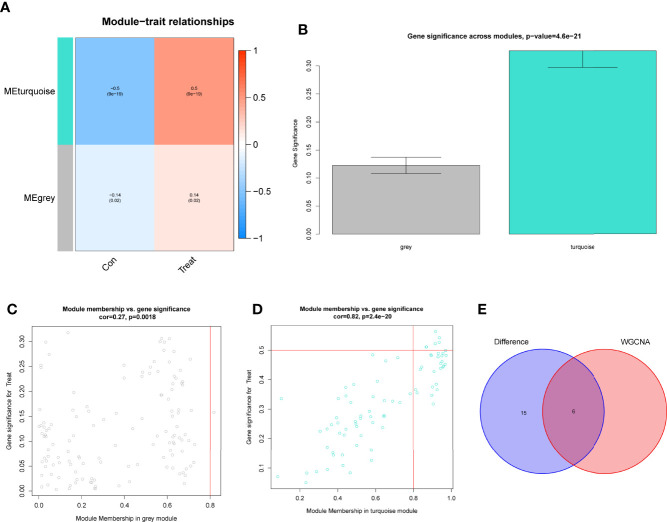
Construction of WGCNA modules. **(A)** He module-trait relationship heat map. RA was strongly linked to the turquoise module. **(B)** Distribution of average gene significance in the modules related to RA. **(C, D)** Associations between module membership and gene importance is depicted in a scatter plot. **(E)** The Overlapping genes between DEGs and the MEturquoise module.

**Figure 6:**
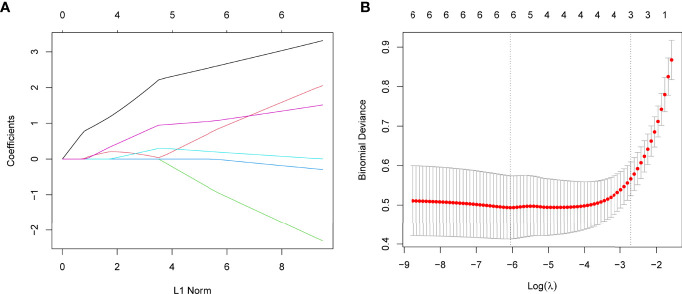
Establishment of diagnostic biomarkers by LASSO regression analysis. **(A) **LASSO coefficient profiles of the six genes in RA. **(B)** The log (lambda) sequence was used to construct a coefficient profile diagram. The LASSO model’s optimal parameter (lambda) was chosen.

**Figure 7:**
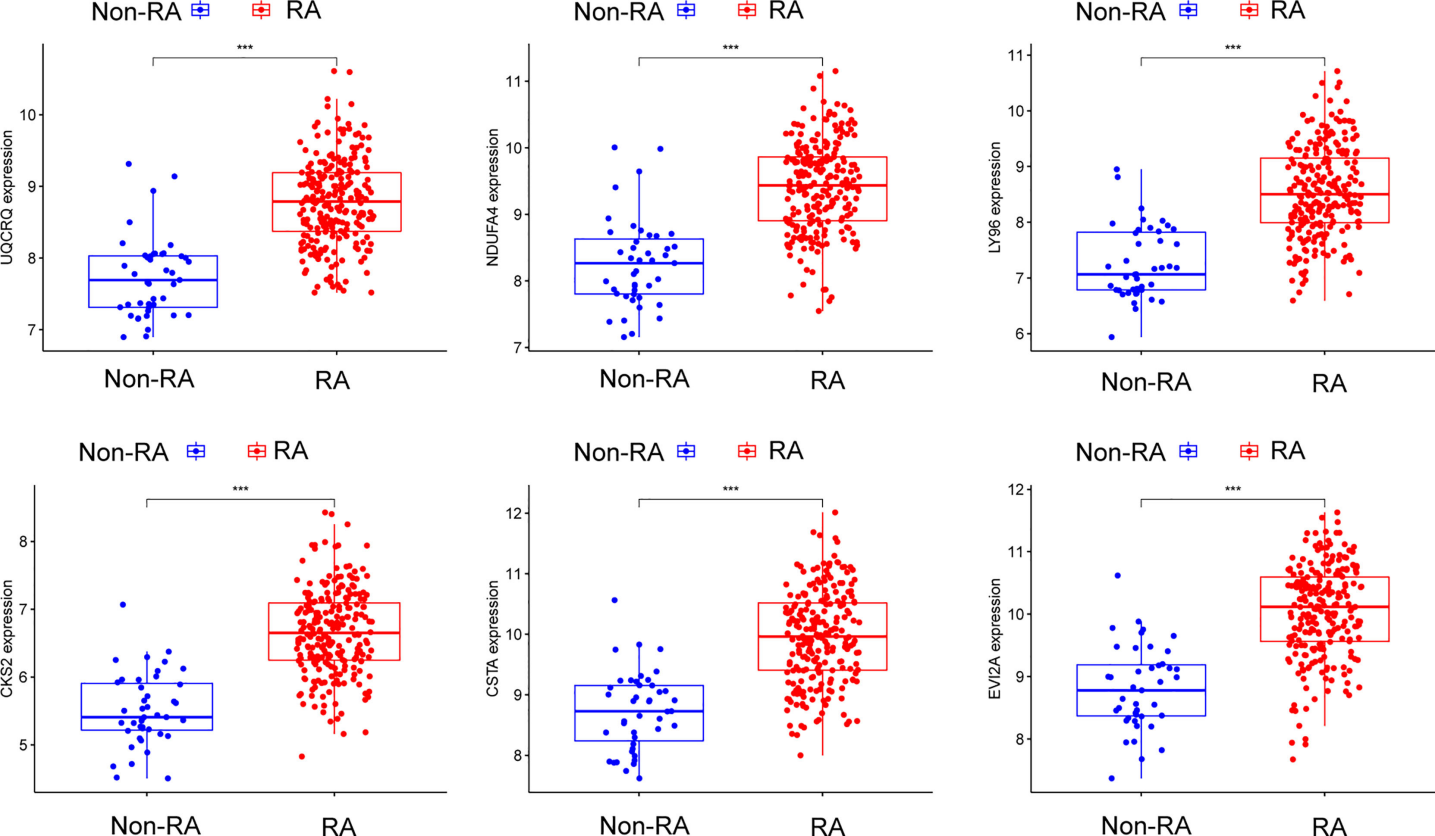
The expressing pattern of six genes in RA samples and normal samples from GSE17755. ***p < 0.001.

**Figure 8:**
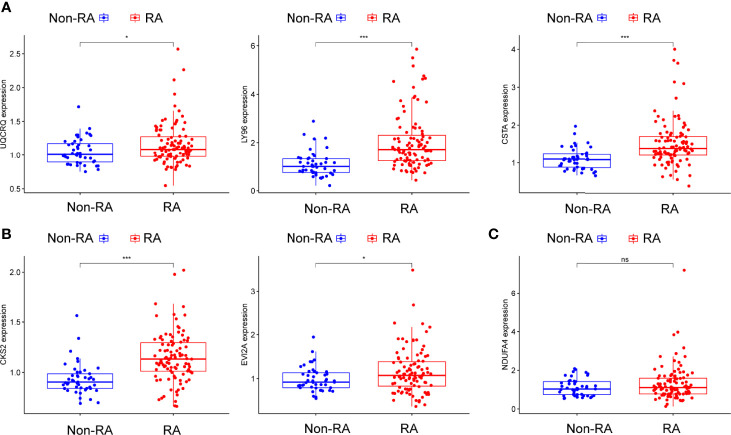
**(A–C) **The expressing pattern of six genes in RA samples and normal samples from GSE93272. *p<0.05, ***p<0.001. ns represents no significance.

**Figure 9:**
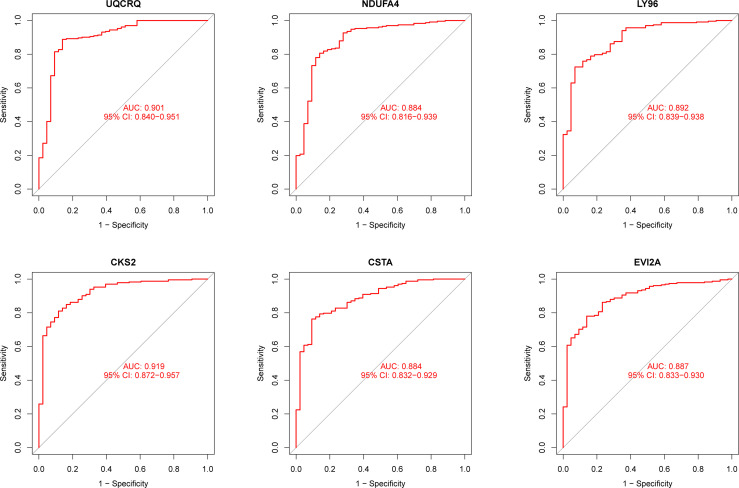
ROC assays for six genes based on GSE17755.

**Figure 10:**
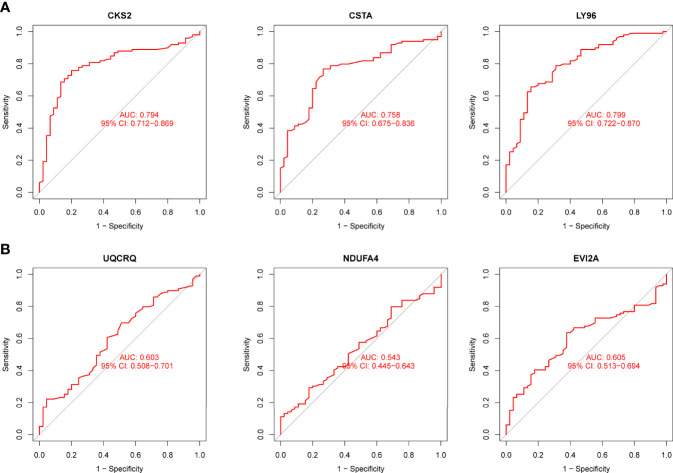
**(A, B)** ROC assays for six genes based on GSE93272.

Using WGCNA analysis, we were able to construct four exceptional coexpression modules. Multiple modules were shown to be associated with RA, as evidenced by the module-trait correlation studies (**Figure 5A**). This data was represented as heat maps, with turquoise (six genes) showing the strongest link to RA of all the modules studied thus far, as well as that of healthy controls (**Figures 5B-D**). Then, six overlapping features (CKS2, UQCRQ, NDUFA4, EVI2A, CSTA and LY96) between the group of DGEs and the group of turquoise were ultimately selected (**Figure 5E**). Moreover, The LASSO regression approach was used to narrow down the six overlapping features, and six variables were identified as diagnostic biomarkers for RA (**Figures 6A, B**). The distinct upregulation of CKS2, UQCRQ, NDUFA4, EVI2A, CSTA and LY96 were observed in RA samples compared with normal samples (**Figure 7**). To further confirm the expressing pattern of the above six genes in RA, we further analyzed GSE93272, and found that only CKS2, UQCRQ, EVI2A, CSTA and LY96 were highly expressed in RA compared with normal samples (**Figures 8A, B**). However, the expression of NDUFA4 remained unchanged between RA samples and healthy samples (**Figure 8C**). Analysis of the AUC values of the six hub genes was used to evaluate their sensitivity and specificity for RA diagnosis in ROC curve analysis. The AUC values of six genes were greater than 0.85, which suggested that these genes were highly diagnostic for RA (**Figure 9**). Using the GSE93272 dataset, the diagnostic usefulness of the six hub genes listed above was further confirmed for clinical purposes. CKS2, CSTA and LY96 had AUC values > 0.75 (**Figure 10A**), whereas the UQCRQ, NDUFA4 and EVI2A had an AUC value <0.7 (**Figure 10B**). Our findings highlighted the potential of CKS2, CSTA and LY96 used as novel diagnostic biomarkers for RA patients.

### Immune Cell Infiltration and Its Associations With Diagnostic Genes

**Figure 11:**
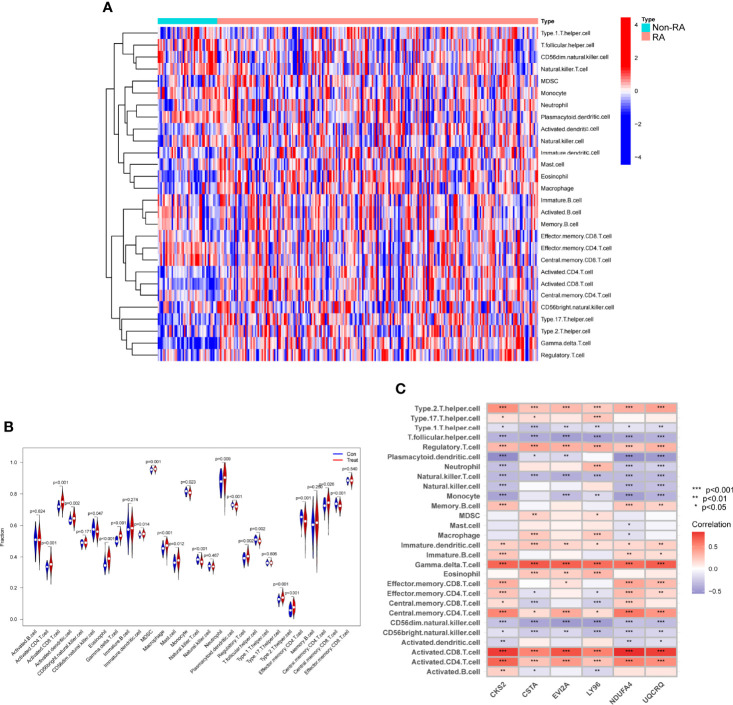
Assays of immune landscape related to RA. Heatmap **(A)** and violin plot **(B)** exhibiting the distribution of 28 immune cells in normal samples and RA samples. **(C)** The associations between immune cell infiltration and six hub genes. *p < 0.05, **p < 0.01, ***p < 0.001.

The ssGSEA algorithm was used to examine the association between RA and healthy controls in terms of differences in immune cell infiltration. **Figure 11A** showed the GSE17755 datasets’ distribution of 28 immune cells. We observed a distinctly higher infiltration of Activated.CD4.T.cell, Activated.CD8.T.cell, Activated.dendritic.cell, Eosinophil, CD56dim.natural.killer.cell, MDSC, Macrophage, Mast.cell, Neutrophil, Regulatory.T.cell, Type.17.T.helper.cell, Type.2.T.helper.cell, Memory.B.cell, Central.memory.CD4.T.cell in RA than in normal specimens, indicating that they play a critical role in developments of RA (**Figure 11B**). Furthermore, correlation analysis confirmed positive correlations of many types of immune cell infiltration with the expression of CKS2, CSTA and LY96 (**Figure 11C**).

### Impact of CKS2 on RA‐HFLS Cell Proliferation

**Figure 12:**
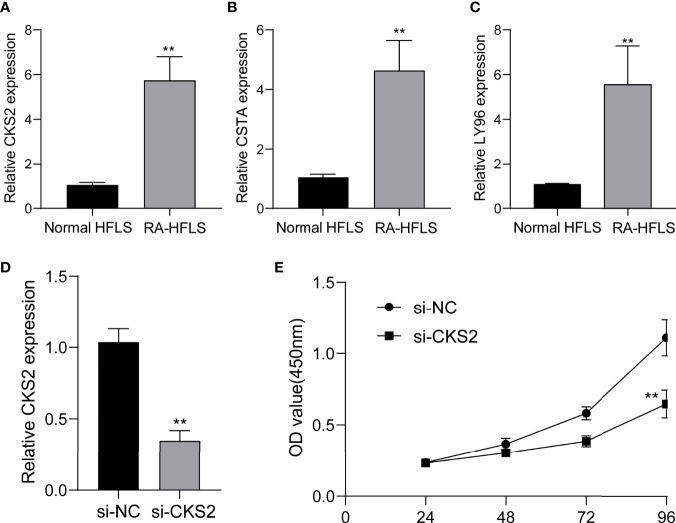
The expression of CKS2, CSTA and LY96 in RA cells and the potential functions. **(A)** CKS2, **(B)** STA, and **(C)** LY96 was highly expressed in RA-HFLS cells compared with normal HFLS cells. **(D)** RT-PCR confirmed the distinct down-regulation of CKS2 in RA-HFLS cells after the transfection of si-CKS2. **(E)** CCK-8 assays revealed that knockdown of CKS2 suppressed the proliferation of RA-HFLS cells.**p < 0.01.

To further demonstrate whether CKS2, CSTA and LY96 exhibited a dysregulated level in RA, we performed RT-PCR and found that the expression of CKS2, CSTA and LY96 was distinctly upregulated in RA-HFLS cells compared with normal HFLS cells (**Figures 12A-C**). Next, we decreased CKS2 expression by the use of siRNA in RA‐HFLS cells. RT-PCR demonstrated the distinct down-regulation of CKS2 in RA‐HFLS cells (**Figure 12D**). In addition, the proliferation of RA‐HFLS upon CKS2 silence were examined by CCK-8. As displayed in **Figure 12E**, knockdown of CKS2 suppressed the proliferation of RA-HFLS cells.

## Discussion

RA is the most commonly diagnosed systemic inflammatory arthritis (26). An untreated RA may exhibit a distinct impact on the quality of life of patients, potentially leading to disability (27). A better understanding of the molecular level of illness detection and treatment is inevitable. Biomarkers that are related with rheumatoid arthritis have been identified. However, the precise mechanism of gene regulation that leads to disease progression has not yet been fully understood (28, 29).

In this study, we analyzed GSE17755 datasets and identified 21 DEGs in RA. Interesting, all 21 DEGs were highly expressed in RA, suggesting them as positive regulator factors in progressions of RA. Then, our group carried out KEGG assays using the 21 DEGs, finding that they were mainly enriched in pathways associated with Ribosome, Chemical carcinogenesis-reactive oxygen species, Coronavirus disease – COVID-19, Oxidative phosphorylation and Huntington disease. Then, we screened 6 possible diagnostic biomarkers for RA, based on WGCNA analysis and LASSO regression algorithm, including CKS2, UQCRQ, NDUFA4, EVI2A, CSTA and LY96. As a data reduction method and an unsupervised classification method, the WGCNA is a hybrid (19). Numerous synthetic gene groups (or modules) are reduced to a handful of easily interpreted gene responses. The use of machine learning-based algorithms in clinical decision-making is widespread (30, 31). Clinical efficacy has been proven for LASSO, one of the most often utilised algorithms. The diagnostic classifier constructed by the LASSO methods and WGCNA has been frequently used in many diseases, such as esophageal cancer, acute coronary syndrome and Sepsis (32–34). However, its application in RA was rarely.

After, we screened six possible biomarkers. Then, we further confirmed their diagnostic using GSE93272 datasets, and further demonstrated CKS2, CSTA and LY96 as critical biomarkers for RA based on the results of ROC assays. Cyclin-dependent kinase regulatory subunits 1 (CKS1) and 2 (CKS2) belong to a family of highly conserved small (9 KDa) cyclin-dependent kinase (CDK)-binding proteins that are involved in the modulation of the cell cycle (35, 36). CKS2 has previously been found to have a significant role in early embryonic developments and somatic cell division (37). However, its function in RA has not been investigated. Similar, the expression and function of CSTA and LY96 in RA also remained largely unclear. In this study, we further used the ssGSEA algorithm to analyze the infiltration of 28 immune cells in RA samples. Compared with normal samples, RA samples had distinctly higher levels of Activated.CD4.T.cell, Activated.CD8.T.cell, Activated.dendritic.cell, Immature.dendritic.cell, Gamma.delta.T.cell, Eosinophil, CD56dim.natural.killer.cell, MDSC, Macrophage, Mast.cell, Neutrophil, Regulatory.T.cell, Type.17.T.helper.cell, Type.2.T.helper.cell, Memory.B.cell, Central.memory.CD4.T.cell. CD8 infiltration in synovial tissues was revealed to be a predictor of RA progression and the existence of antibodies against citrullinated peptides by one investigation (38, 39). Moreover, our group found that the expressions of CKS2, CSTA and LY96 were related to the levels of many immune cells, highlighting their potential used as therapeutic targets for RA.

Finally, we performed RT-PCR to confirm the expressions of CKS2, CSTA and LY96 in RA-HFLS cells and normal HFLS cells. Our findings were consistent with the results from GEO datasets. The levels of CKS2, CSTA and LY96 were distinctly upregulated in RA-HFLS cells compared with normal HFLS cells. Moreover, we decreased the CKS2 expressions by introducing si-CKS2 or their NC cells into RA‐HFLS. Then, the results of CCK-8 assays revealed that knockdown of CKS2 distinctly suppressed the proliferation of RA-HFLS cells. Our findings further demonstrated CKS2 as a therapeutic target for RA.

Although we integrated a number of bioinformatics approaches and statistical methodologies, and performed diverse studies to uncover the diagnostic biomarkers, significant limitations should be noted. Firstly, this was a retrospective study, and thus it lacked new clinical samples and data. Secondly, the biological activities of the identified genes and the connections between those genes and RA have not been completely researched. Finally, the analysis relies solely on GEO databases. To support our findings, we would benefit from additional data from other sources.

## Conclusion

Overall, we integrated multiple bioinformatics tools and identified three critical diagnostic genes in RA. In addition, three critical diagnostic genes infiltrating the immune microenvironment were identified in this research, which could function as novel markers and immune therapeutic targets. However, Further research is needed to support our findings that they may act as therapeutic targets for RA.

## Data Availability Statement

The datasets presented in this study can be found in online repositories. The names of the repository/repositories and accession number(s) can be found below: https://www.ncbi.nlm.nih.gov/, GSE17755, https://www.ncbi.nlm.nih.gov/, GSE93272.

## Author Contributions

Conception, FJ and HS. Design and revision of the manuscript, FJ and HZ. Analysis and interpretation of data, FJ and HS. All authors contributed to the article and approved the submitted version.

## Funding

This work was supported by National Natural Science Foundation of China (No. 81960302), Gansu Province Clinical Research Center for Rheumatology(21JR7RA437), Cuiying Scientific and Technological Innovation Program of Lanzhou University Second Hospital (No. CY2021-BJ-A01).

## Conflict of Interest

The authors declare that the research was conducted in the absence of any commercial or financial relationships that could be construed as a potential conflict of interest.

## Publisher’s Note

All claims expressed in this article are solely those of the authors and do not necessarily represent those of their affiliated organizations, or those of the publisher, the editors and the reviewers. Any product that may be evaluated in this article, or claim that may be made by its manufacturer, is not guaranteed or endorsed by the publisher.
